# Effect of Tea Tree Oil on the Expression of Genes Involved in the Innate Immune System in Goat Rumen Epithelial Cells

**DOI:** 10.3390/ani11082460

**Published:** 2021-08-21

**Authors:** Zixuan Hu, Miao Lin, Xiaoyu Ma, Guoqi Zhao, Kang Zhan

**Affiliations:** College of Animal Science and Technology, Yangzhou University, Yangzhou 225009, China; huzixuan2021666@163.com (Z.H.); linmiao@yzu.edu.cn (M.L.); maxiaoyu0417@163.com (X.M.); gqzhao@yzu.edu.cn (G.Z.)

**Keywords:** goat rumen epithelial cells, tea tree oil, LPS, innate immune

## Abstract

**Simple Summary:**

Subacute rumen acidosis (SARA) often causes significant losses on commercial farms. SARA is mainly caused by endotoxin (LPS) produced by the lysis of Gram-negative bacteria, which causes an inflammatory response. To alleviate the inflammatory response mediated by LPS, it is important to improve animal production performance. Tea tree oil (TTO) is a plant extract that possesses good bactericidal and anti-inflammatory effects. According to this study, LPS can significantly induce inflammatory responses in goat rumen epithelial cells (GRECs), while the addition of TTO could markedly mitigate inflammatory responses mediated by LPS in GRECs. Therefore, it may be useful for the treatment of SARA.

**Abstract:**

In subacute rumen acidosis (SARA), the rumen epithelium is frequently attacked by endotoxin (LPS), which is caused by the lysis of dead Gram-negative bacteria. However, the rumen epithelium innate immune system can actively respond to the infection. Previous studies have demonstrated that tea tree oil (TTO) has good bactericidal and anti-inflammatory effects. Therefore, the aim of this study was to investigate the effect of TTO on the expression of genes involved in the inflammatory cytokines in goat rumen epithelial cells (GRECs) triggered by LPS. Our study shows that rumen epithelial cells isolated from goat rumen tissue can be cultured in vitro in 0.25% trypsin for a long time. These cells were identified as epithelial cells by the expression of cytokeratin 18, monocarboxylate transporter 4 (MCT4), Na[+]/H[+] hydrogen exchanger 1 (NHE1), putative anion transporter 1 (PAT1), vH^+^ ATPase B subunit (vH^+^ ATPase), and anion exchanger 2 (AE2). The mRNA expression of IL-1β, IL-6, TNF-α, TLR-2, NF-κB, CXCL6 and CXCL8 genes was significantly increased when LPS was used compared to untreated controls. In addition, mRNA expression of IL-1β, IL-6, TNF-α, TLR-2, NF-κB, CXCL8, CXCL6 and interferon-induced protein with tetratricopeptide repeats 3 (IFIT3) genes was also significantly higher in the LPS group compared to the 0.05% TTO group. However, the expression of IL-1β, IL-6, TNF-α, TLR-2, CXCL6 and IFIT3 genes was significantly lower in the LPS and 0.05% TTO group compared to the 1 μg/mL LPS group. These results suggest that TTO can inhibit LPS-induced inflammatory cytokines expression in GRECs.

## 1. Introduction

Subacute rumen acidosis (SARA) is a common disease in commercial farms, causing huge economic losses [1,2]. SARA is a metabolic disease caused by excessive intake of high fermented carbohydrates and insufficient dietary fiber in ruminants [3]. In the rumen, a highly fermentable diet is quickly converted into organic acids [4]. The accumulation of single-chain fatty acids causes bacterial disintegration which generates LPS that disrupts the integrity of the rumen epithelium, leading to SARA, and inducing a cascade of inflammatory responses [5]. Therefore, the establishment of a goat rumen epithelial cells (GRECs) line is of great importance for further investigation of the expression of goat genes involved in SARA-related pro-inflammatory cytokines. Goats with SARA experience certain stressful states and increased free LPS in the rumen [6,7]. Higher levels of LPS in rumen fluid may lead to localized inflammation of rumen epithelium [8], while LPS induces innate immune response via Toll-like receptors (TLR) [9]. TLR-2 and TLR-4 are the major recognition receptors for LPS identified so far out of 10 Toll-like receptors [10,11]. Among the many signaling pathways that respond to inflammatory responses, the nuclear factor-κB (NF-κB) pathway is an important signaling pathway in response to LPS [12]. The NF-κB is located downstream of the TLR signaling pathway and plays a pivotal role in the regulation of immune and inflammatory responses [13]. The LPS activates the NF-κB inflammatory signal pathway, and further promotes the expression of inflammatory factors, including interleukin 1 beta (IL-1β), tumor necrosis factor alpha (TNF-α) and interleukin 6 (IL-6) [14]. In addition, myeloid differentiation primitive-response protein 88 (MyD88) is also an essential molecule induced by LPS in a typical NF-κB signaling pathway [15,16]. In previous studies, some endogenous regulatory factors have been identified that specifically regulate TLR signaling pathways. Among them, Toll-interacting protein (Tollip) is a key regulator of TLR-mediated innate immune response [17]. In the innate immune response, Tollip can inhibit TLR-mediated cellular responses by binding to TLR-2 or TLR-4 [18]; it also negatively regulates IL-1β and TNF- α signaling pathways [19], and overexpression of Tollip impairs NF-κB downstream signaling [20]. As mentioned above, LPS plays an important regulatory role in the process of inducing inflammatory responses within rumen tissues. Therefore, strategies to reduce inflammation of the rumen epithelium are crucial. The drugs currently used to inhibit inflammation sometimes have unpleasant side effects. Consequently, the search for a plant extract that alleviates epithelial inflammation with low toxic effects is vital to animal welfare.

Tea tree oil (TTO) is an essential oil extracted from the leaves of the tea tree, native to the Melaleuca Alternifolia tree in Australia [21]. TTO has long been used by Australian Aborigines to treat diseases related to inflammation [22]. TTO contains about 100 compounds, mainly monoterpenes, sesquiterpenes and their related alcohols [23,24]. Previous research found that TTO has a significant inhibitory effect on inflammation in vitro. The water-soluble component of TTO significantly inhibited LPS-induced expression of IL-10, IL-1β and TNF-α by human peripheral blood mononuclear cells [25]. TTO is mainly composed of α-terpineol, terpinen-4-ol, and 1,8-cineole, but only terpinen-4-ol can inhibit the secretion of cytokines such as IL-1β, TNF-α, and IL-10 from LPS-stimulated human monocytes [26]. Interestingly, the mechanism of action of terpinen-4-ol and α-terpineol does not interfere with cytokine production, but instead inhibits superoxide production [27,28]. In addition, further studies found that TTO reduced the production of reactive oxygen species in stimulated neutrophils and monocytes [29]. In epithelial cells, TLR is activated by their interacting ligands, releasing a series of cytokines, antimicrobial peptides and chemokines which trigger inflammatory and immune responses to eliminate pathogens, thus helping to maintain the function of the epithelial barrier [30]. NF-κB signaling is specifically associated with the expression of inflammatory cytokines after TLR activation [31]. Thus, inhibition of NF-κB activation interferes with the cytokine network and thus regulates inflammatory diseases. Studies have shown that TTO can inhibit inflammatory mediator production in LPS-stimulated human macrophages by interfering with the NF-κB, extracellular signal-regulated kinases (ERK) and p38 mitogen-activated protein kinase (MAPK) pathway [32]; however, there is no report on whether TTO has an anti-inflammatory effect in LPS-stimulated goat rumen epithelial cells.

The primary objective of the present study was to establish a stable passage GRECs line and study goat rumen nutrient absorption and regulation. The second objective of the study was to investigate the regulatory effects of TTO on the expression of genes associated with the inflammatory cytokines and immune signal pathway in GRECs induced by LPS.

## 2. Materials and Methods

### 2.1. Isolation and Cultivation of Primary Goat Rumen Epithelial Cells

Goat rumen epithelial cells (GRECs) were obtained from rumen tissue isolated from three 60-day-old healthy goats. The animals used in this study were in accordance with the guidelines of the Institutional Animal Care and Use Committee (IACUC) of Yangzhou University. Primary GRECs were obtained as described previously with minor adjustments [33]. After anesthetizing the goats, a sterile procedure was performed to split open the abdominal cavity to remove the rumen. After washing with sterile saline, the tissues were immediately rinsed repeatedly with phosphate buffer containing 500 U/mL penicillin, 250 μg/mL gentamicin, 500 μg/mL streptomycin, and 12.5 μg/mL amphotericin B (5× PSGA; Invitrogen, Shanghai, China). The tissue was placed in DMEM medium (Invitrogen) containing 5× PSGA and placed in a 37 °C oscillator and slowly shaken for 30 min. The mucosa of the rumen epithelium was separated with ophthalmic scissors and forceps, then cut into approximately 1 mm^3^-sized pieces and centrifuged at 200× *g* for 1 min at 4 °C until the supernatant was clear. The chopped epithelium was transferred into a 40 mL digestion flask containing 0.25% trypsin-0.02% EDTA for digestion. The flask was shaken and digested for 10 min at 37 °C in an air bath. After digestion, the flask was centrifuged for 5 min at 1000 r/min and the supernatant was removed (try to blow and aspirate to promote separation of individual cells) and repeated 3 to 5 times. The resulting digest was filtered through a 74 μm sieve into a 50 mL sterile tube and a DMEM medium containing 10% FBS was added. The sterile tube was centrifuged at 200× *g* for 5 min at 4 °C and the supernatant was taken. The cell suspension was washed 3 times with DMEM medium containing 5× PSGA. Afterwards, a DMEM medium containing 10% FBS, 100 U/mL penicillin, 100 μg/mL streptomycin, 4 mm/L glutamine, 1% non-essential amino acids, 1× Insulin-Transferrin-Selenium (1× ITS; Invitrogen, Waltham, MA, USA), 15 ng/mL epidermal growth factor (PeproTech, Shanghai, China) was resuspended and diluted to 2 × 10^5^ cells/mL and inoculated in 6-well culture plates. Each well was incubated at 37 °C with 5% CO_2_ saturated humidity. The GREC may take up to 72 h to complete attachment therefore plates were left unagitated during this period.

### 2.2. Proliferative Activity Analysis

The 2nd generation GRECs were taken into 96-well plates and inoculated with about 5 × 10^2^ cells/well. Twenty-four hours later, 20 μL of CCK8 solution was added to each well, and the culture was further incubated for about 2 h. The 96-well plates were then placed in an automated microplate reader (Thermo Scientific, Shanghai, China), and the absorbance of each well was measured at 450 nm for 7 consecutive days to plot the growth curve.

### 2.3. Cell Senescence Analysis

The primary GRECs were inoculated in 6-well plates. These primary GRECs were washed with PBS after 24 h of incubation. According to the staining protocol (Biyuntian, Beijing, China), 1 mL of β-galactosidase staining fixative was added to the cells and fixed at room temperature for 15 min. Subsequently, the fixative was discarded and the cells were washed three times with PBS for 3 min each. After washing, l mL of staining solution was added to each well, incubated overnight at 37 °C, and then sealed with plastic wrap to prevent evaporation. Finally, the cells were observed under the microscope.

### 2.4. Immunofluorescence Analysis

Cells cultured on a chamber slide were fixed with 4% paraformaldehyde at 4 °C for 30 min. The slides were then washed 3 times with PBS for 3 min each time. PBS containing 3% horse serum was added dropwise to the slides and blocked at room temperature for 1 h. Blocking solution was removed, mouse-derived cytokeratin 18 primary antibody (1:1000; Abcam, Shanghai, China) was then added and incubated overnight at 4 °C. Next, anti-mouse IgG conjugated to FITC (1:400; Santa Cruze, Shanghai, China) was added and incubated for 1 h at room temperature and protected from light. The cultured cells were washed with PBS 3 times and then DAPI staining solution was added. After staining for 8 min, the cells were washed 3 times with PBS, and 1 time with distilled water. Finally, a confocal laser scanning microscope (Olympus, Tokyo, Japan) was used for visualizing cells.

### 2.5. Identification of Goat Rumen Epithelial Cells

To verify the origin of the cultured GRECs, RT-PCR was performed to detect the expression of transporter and receptor proteins involved in SCFAs, including the monocarboxylate transporter 4 (MCT4), Na[+]/H[+] hydrogen exchanger 1 (NHE1), Putative anion transporter 1 (PAT1), vH^+^ ATPase B subunit (vH^+^ ATPase), and Anion exchanger 2 (AE2). Total RNA was extracted with TRIzol (Invitrogen, Beijing, China). Total RNA was reverse transcribed to cDNA according to the Takara Reverse Transcription Kit, and the test was operated on ice with a reverse transcription system of 10 μL: total RNA 500 ng, 5× PrimeScript RT Master Mix 2 μL and RNAase-free water. The RT reaction was performed at 37 °C for 15 min, heated to 85 °C and continued for 5 s. The PCR reaction was performed with SYBR^®^ Premix Ex Taq™ II Kit (Takara, Dalian, China). The reaction system consisted of SYBR^®^ PreMix Ex Taq™II 10 μL, 10 μM forward primer, 10 μM reverse primer, and 100 ng cDNA templates in a final volume of 20 μL. The reaction conditions were 95 °C pre-denaturation for 30 s, followed by 40 cycles at 95 °C for 5 s and 60 °C for 30 s. The PCR products were separated by 1% agarose gel electrophoresis and subsequently observed in a gel imager. The primers are listed in Table 1. Primer design was derived from previous studies and GAPDH was also selected as an internal control gene based on previous work on bovine cells [34,35].

### 2.6. Experimental Design and Treatment

The GRECs were inoculated to a certain density in 6-well plates with 1 × 10^5^ cells per well and cultured for 12 h. The culture continued after the following treatments: the control group was supplemented with DMEM/F12 medium only; the TTO group was supplemented with DMEM/F12 medium and 0.05% TTO; the LPS group was supplemented with DMEM/F12 medium and 1 μg/mL LPS; the LPS + TTO group was supplemented with 1 μg/mL LPS and 0.05% TTO. Each group had 6 replicates. After 24 h of incubation, the cells of each group were collected separately, and total RNA was extracted. Tea tree oil extracts were obtained from Wuxi Chenfang Biotechnology Co., Ltd. (Wuxi, Jiangsu, China).

### 2.7. qRT-PCR

Total cellular RNA was extracted using TRIzol reagent (Invitrogen, Shanghai, China). Reverse transcription was performed using RT Kit (Takara, Dalian, China). The total reverse transcription system was 20 μL. Reaction conditions: 37 °C for 15 min, 85 °C for 5 s. The SYBR^®^ Premix Ex Taq™ II kit (Takara, Dalian, China) was used for qRT-PCR reaction. The qRT-PCR reaction system (20 μL) was as follows: 2× SYBR^®^ Premix Ex Taq™ II, forward primer 10 μM, reverse primer 10 μM, and 100 ng cDNA template in a final volume of 20 μL. The qRT-PCR reactions were initially denatured at 95 °C for 30 s, followed by 40 cycles at 95 °C for 5 s and 60 °C for 30 s. The primers used are shown in Table 1. The relative expression of target genes with GAPDH expression was normalized and calculated by the 2^−ΔΔCT^ method.

### 2.8. Statistical Analysis

Before data analysis, Kolmogorov−Smirnov and Levene tests were used to study the distribution of normality and variance uniformity. The data conformed to normal distribution and homogeneity of variance, and one-way ANOVA statistical analyses were performed using SPSS 19.0 statistical package software (SPSS Inc., Chicago, IL, USA) to determine the lowest significant difference in multiple comparisons after treatment of the means. The parametric test was used for statistical analysis. The gene expression for the 2^−ΔΔCt^ were log_10_ transformed for statistical analysis. *p* < 0.05 was considered significant, and *p* < 0.01 was considered highly significant. Trends toward significance are discussed at 0.05 < *p* < 0.10.

## 3. Results

### 3.1. Characterization of the Morphology of Primary Goat Rumen Epithelial Cells

**Figure 1:**
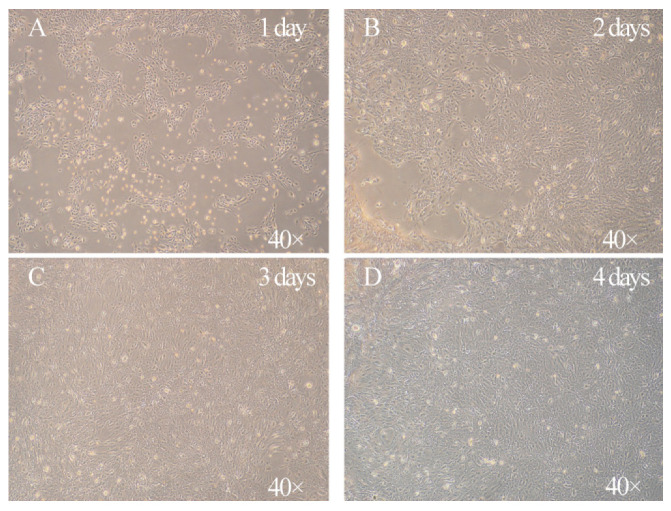
The morphology of cultured GRECs. (**A**) 1 day of culture. (**B**) 2 day of culture. (**C**) 3 day of culture. (**D**) 4 day of culture. Magnification 40×.

**Figure 2:**
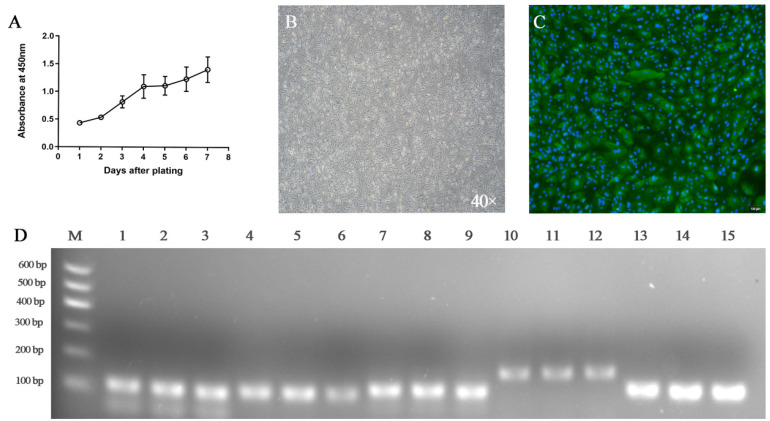
Characteristics of goat rumen epithelial cells. (**A**) Growth curve of GRECs in culture. Data shown are means ± SEM (*n* = 3). (**B**) Analysis of GRECs senescence (100×). The appearance of blue is due to the expression of β-galactosidase, which indicates that senescence occurs in GRECs. (**C**) Immunoblotting of cytokeratin-18. Indirect immunofluorescence of cytokeratin 18 (green). Nuclei were stained with DAPI (blue). Bar = 100 μm. (**D**) RT-PCR analysis of cell markers in GRECs. RT-PCR analysis of MCT4, NHE1, PAT1, vH^+^ ATPas, and AE2; amplifications of GREC-specific markers were obtained using primers directed against (lanes 1–3) MCT4, 106 bp, (lanes 4–6) NHE1, 113 bp, (lanes 7–9) PAT1, 123 bp, (lanes 10–12) vH^+^ ATPas, 182 bp, and (lanes 12–15) AE2, 123 bp. M molecular weight standard in bp. The data were based on triplicate experiments.

In this study, we isolated primary GRECs from goat rumen tissue. The isolated GRECs had typical epithelial and cobblestone morphology characteristics (Figure 1). After 2 day of culture, the phenomenon of cell adhesion was observed under light microscope, most of which were flat and polygonal, and the shapes were clear (Figure 1B). These cells density can reach 100% confluence after 4 days of culture (Figure 1D). The growth curve of GRECs showed that after an initial delay of 2 day, the cells entered the logarithmic growth phase (days 2 to 4). Growth was slow on the 5th day, however, there was a relatively rapid growth on the 6th and 7th day (Figure 2A). Notably, there was some β-galactosidase expression in primary GRECs, which indicated senescence signals (Figure 2B).

### 3.2. Characterization of Goat Rumen Epithelial Cells

It is well known that cytokeratin 18 is a unique marker protein on the surface of epithelial cells, which can be used to identify whether isolated cells are epithelial cells. Therefore, cytokeratin 18 can be used to identify whether the primary GRECs come from epithelial cells. The immunofluorescence results showed that green fluorescence was emitted around the detected cells (Figure 2C), which proved that the isolated cells express cytokeratin 18. The results showed that the isolated primary GRECs originated from epithelial morphology. To further evaluate the expression of GRECs marker genes, MCT4, NHE1, PAT1, vH^+^ ATPas, and AE2 were analyzed using RT-PCR. These genes are considered to be specific SCFAs transporters on GRECs. RT-PCR analyses confirmed the presence of MCT1, NHE1, PAT1, vH^+^ ATPas, and AE2 bands were observed in primary GRECs (Figure 2D). These results proved that GRECs are originated from rumen epithelial cells.

### 3.3. Effect of TTO on LPS-Induced Inflammatory Cytokine Expression

**Figure 3:**
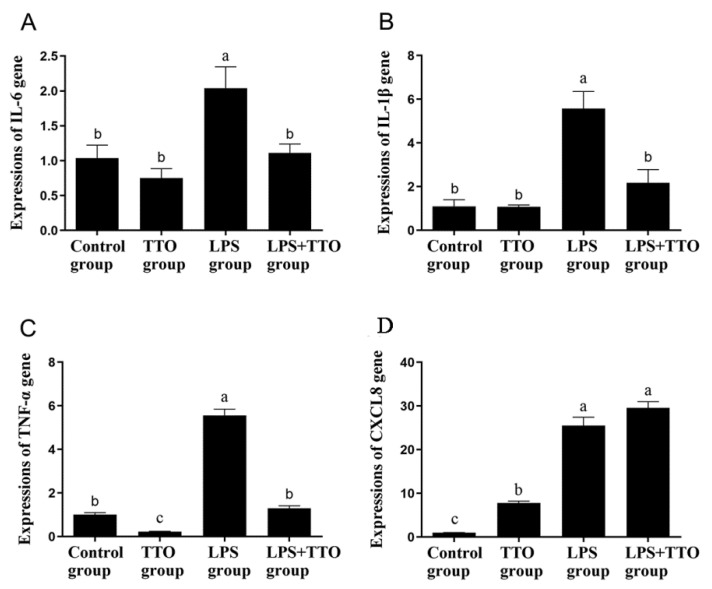
Effect of TTO on gene expression of inflammatory cytokines in GRECs. Graphs illustrating levels of IL-6 (**A**), IL-1β (**B**), TNF-α (**C**), and CXCL8 (**D**) are shown. GAPDH was used as an internal reference gene. Data shown are means ± SEM (*n* = 3). Different lowercase letters indicate significant differences (*p* < 0.05).

The expression of IL-6, IL-1β, CXCL8 and TNF-α was significantly upregulated in the LPS group compared with the control and TTO groups (Figure 3, *p* < 0.05). However, compared with the LPS group, the expression of inflammatory cytokines IL-6, IL-1β and TNF-α was significantly lower in the TTO + LPS group (Figure 3A–C, *p* < 0.05), while the expression of CXCL8 was not significantly changed (Figure 3D, *p* > 0.05).

### 3.4. Effect of TTO on LPS-Induced Innate Immune Responses

The results of the quantitative PCR performance associated with the inflammatory response of GRECs are displayed in Table 2. LPS significantly promoted the expression of inflammation-regulated genes TLR-2, NF-κB and CXCL6 in GRECs compared with the control and TTO groups (*p* < 0.001). In addition, compared with the control group, TTO did not affect other genes except for downregulating TLR-2 expression. The expression of TLR-2, IFIT3 and CXCL6 was significantly reduced in the TTO + LPS group compared with the LPS group (*p* < 0.05). Interestingly, the expression of TLR-4 and IRF3 in the TTO + LPS group was significantly higher than that in the LPS group (*p* < 0.05). Remarkably, the expression of CCL5, MYD88 and TOLLIP was not affected by any added reagents (*p* > 0.05).

## 4. Discussion

In the present study, we established and validated primary GRECs, and investigated the effect of TTO on LPS-induced GRECs inflammatory factor expression. The rumen epithelium is responsible for many important physiological functions, including nutrient absorption, immune barrier, and metabolism of SCFAs [36]. However, the application of rumen epithelial cell culture technology in goats is still rare, and worst of all, the cultured rumen epithelial cells can only be passaged for a limited time, after which the cells appear to undergo senescence and apoptosis. In order to establish a stable passage GRECs line, we used 0.25% trypsin for separation and digestion to obtain the primary GRECs and established a stable passage of more than 20 generations. To verify the origin of GRECs lines, we first performed immunocytochemical analysis of cytokeratin 18. Cytokeratin is commonly used to distinguish epithelial cells from fibroblasts [33]. Cytokeratin is the backbone protein of epithelial cells; however, fibroblasts are not capable of expressing cytokeratin and therefore, epithelial cells cannot be identified by cytokeratin [37]. Our results showed that the nucleus fluoresces blue after DAPI staining and cytokeratin 18 fluoresces green which implied that the current cultured cells are of epithelial cell type. In addition to this, we demonstrated that the GRECs line can express MCT4, NHE1, PAT1, vH^+^ ATPas, and AE2. The data on sheep show that up to 50% of SCFA can be absorbed through the exchange of SCFA^-^ with HCO^3−^ [38,39]. The PAT1 and AE2 are the main exchange carriers between SCFA^-^ and HCO^3−^, and both proteins are expressed in rumen epithelial cells [40]. In addition to the above mentioned SCFA^-^ and HCO^3−^ exchange carriers, the monocarboxylate transport carrier (MCT) family is also an important route for SCFA to enter the rumen epithelium. The MCT family includes 14 members, but studies have shown that only MCT 1−4 can catalyze the cotransport of monocarboxylate molecules with H^+^ [41,42]. MCT 4 is mainly distributed in the top of rumen epithelium (stratum corneum and granular layer), and many experiments have demonstrated that it can be directly involved in the uptake of SCFA by the rumen epithelium [43,44]. The pH-regulated related proteins also play an important role in the uptake of SCFA by the rumen epithelium. The NHE1 is an important transmembrane protein that regulates intracellular pH by exchanging one intracellular H^+^ with one extracellular Na^+^ in equal molecular proportions across the membrane, thereby expelling intracellular H^+^ which causes an increase in intracellular Na^+^, and the NHE1 has been shown to be expressed in the rumen epithelium [45,46]. The VH^+^ ATPas is mainly distributed in the spinous process layer and granular layer of the rumen epithelium, and about 30% of the H^+^ excretion in goat rumen epithelial cells is accomplished by vH^+^ ATPas [47,48]. In the rumen, 50–85% of the SCFA produced is absorbed by the rumen epithelium; afterward, the rest enters the subsequent intestinal segment [49]. Therefore, SCFA transport and uptake are the most important functions of rumen epithelial cells. Our results show that genes for SCFA transporter carriers (MCT4, PAT1 and AE2) and pHi regulatory proteins (NHE1 and vH^+^ ATPas) can be expressed in GRECs lines, suggesting that our cultured cells are capable of transporting and taking up SCFA. These results demonstrated that the primary GRECs line originates from goat rumen epithelial cells.

Inflammation of the rumen is a direct symptom of SARA. The mechanism of inflammation is that low rumen pH leads to the death and lysis of Gram-negative bacteria which increases the concentration of free LPS in the rumen, which triggers inflammation [50]. Supplementation of plant essential oils represents a commonly used approach to mitigate the consequences of SARA. Some studies have proven that cinnamon oil, thyme oil, and garlic oil are all helpful in relieving SARA [51,52,53]. However, there are no reports of TTO treatment for SARA. The water-soluble components of TTO, especially 4-terpinenol, contribute to the inhibition of superoxide ions in monocytes and the production of inflammatory mediators (e.g., TNF-α, IL-1β, IL-8, IL-10 and PGE2) [25,54]. This limits further production of other inflammatory cytokines and reduces oxidative damage to cells [55]. Previous studies have demonstrated the anti-inflammatory effects of TTO [56]; however, the effect of TTO in modulating the innate immune response of the rumen epithelium induced by LPS is unknown. The LPS firstly binds to LPS-binding protein (LBP) to form the LPS-LBP complex, which significantly enhances the biological activity of LPS [57]. The LPS-LBP complex is recognized and bound by CD14 and myeloid differentiation protein 2 (MD2) receptors on the cell surface. The CD14 then delivers the LPS-LBP complex to the TLR, which activates the MyD88-dependent signaling pathway and the TLR structural domain bridging protein (TRIF)-dependent signaling pathway, thereby triggering the MAPK/NF-κB signaling pathway [58,59]. The activated NF-κB enters the nucleus and interacts with the promoters of related genes, thus promoting the release of inflammatory factors or chemokines, such as TNF-α, IL-1β, IL-6 and CXCL8, which trigger inflammatory responses [59,60]. High expression of TNF-α can induce activation of neutrophils, further causing massive expression of other pro-inflammatory factors and activation of inflammatory signaling molecules [61]. The IL-1β is a mediator of acute and persistent inflammation [62]. The IL-6 is a pleiotropic protein that has a strong effect on the inflammatory response and is a major factor in the acute phase response [63]. The CXCL8 is a leukocyte chemotactic-activated cytokine produced by a variety of cells in response to inflammatory stimuli and has a cytotactic effect on neutrophils to achieve its regulation of the inflammatory response [64]. The results showed that the expression of IL-1β, IL-6, CXCL8, TNF-α, TLR-2 and NF-κB was significantly increased after LPS infested GRECs, but did not affect the expression of TLR-4 gene. This suggests that LPS enhances pro-inflammatory factor expression through activation of the NF-κB pathway by TLR-2 ligands to induce the proinflammation response. Compared with LPS alone, TTO decreased the expression of IL-1β, IL-6, TNF- α, TLR-2 and NF-κB in GRECs. This result demonstrated that the use of TTO attenuates the LPS-induced pro-inflammatory response in GRECs. This implies that TTO can inhibit the NF-κB signaling pathway during the onset of the inflammatory response, blocking IL-1 β, IL-6, TNF- α and TLR-2 overexpression, thereby providing relief from LPS-induced inflammation. When pathogens invade, GRECs trigger the activation of the innate immune response through the induction of chemokines and adhesion molecules [65]. The main role of chemokines is to converge a large number of immune cells to the point of tissue localization, and these cells secrete various active products such as pro-inflammatory cytokines, oxygen free radicals, matrix metalloproteinases, etc., which are involved in immune damage and inflammatory response of the tissue [66]. The IFIT3 is one of the representative genes of the type I interferon system, and it has been shown that IFIT3 positively regulates the expression of chemokines [67]. Our results showed that chemokines CXCL8 and CXCL6 were significantly upregulated in the LPS group compared to the control group. This suggests that LPS can promote the expression of chemokines, and the increased expression of chemokines can further chemotactic immune cells to the ruminal immune layer to process the LPS. In contrast, CXCL6 and IFIT3 expression was decreased in the TTO and LPS + TTO groups relative to the LPS group. It was shown that TTO could alleviate the toxic effects caused by LPS and regulate the expression of chemokines by inhibiting the expression of IFIT3 in GREC. The above results can indicate that TTO can alleviate the stimulation of LPS and can inhibit the activation of NF-κB signaling pathway in the organism, thus acting as an anti-inflammatory function. Hence, our results suggest that 0.05% TTO can inhibit the LPS-induced inflammatory response in the rumen epithelium. These results suggest that TTO may be beneficial for the treatment of SARA.

## 5. Conclusions

In this study, primary GRECs were isolated by digesting goat rumen epithelial tissue with 0.25% trypsin. These primary GRECs can be stably passaged while maintaining morphological characteristics. The cytokeratin 18, MCT4, NHE1, PAT1, vH^+^ ATPas, and AE2 were positive in primary GRECs, suggesting that the cultured cells are rumen epithelial cells. Our study also revealed that TTO can inhibit LPS-induced inflammatory factor expression in GRECs, which provides new ideas for the treatment of SARA in ruminants.

## Figures and Tables

**Table 1:** Primers for real-time PCR analyses.

Gene	Primer Sequence, 5′ to 3′	Accession Number	Product Size (bp)
MCT4	F: GTTTGGGATAGGCTACAGTGACACA	NM_001109980.1	106
	R: GCAGCCAAAGCGATTCACA		
NHE1	F: CCTCTACAGCTACATGGCCTAC	Etschmann et al. (2006)	113
	R: GGGAGATGTTGGCTTCCA		
PAT1	F: CCTTGAGGCACGGCTAC	BC123616.1	123
	R: GCACCAGACTCCGAGACATA		
AE2	F: AGCAGCAACAACCTGGAGT	NM_001205664.1	123
	R: GGTGAAACGGGAGACGAA		
vH^+^ ATPas	F: TTTTATTGAACAAGAAGCCAATGA	Albrecht et al. (2008)	182
	R: GATTCATCAAATTGGACATCTGAA		
IL-1β	F: GAAGAGCTGCACCCAACA	XM_013967700.2	172
	R: CAGGTCATCATCACGGAAG		
IL-6	F: AGATATACCTGGACTTCCT	NM_001285640.1	80
	R: TGTTCTGATACTGCTCTG		
TNF-α	F: TGGTTCAGACACTCAGGT	NM_001286442.1	75
	R: CGCTGATGTTGGCTACAA		
CXCL8	F: TGTGTGAAGCTGCAGTTCTGT	XM_005681749	186
	R: TGGGGTCTAAGCACACCTCT		
CCL5	F: GTCTGCCTCCCCATATGCCTC	XM_005693201	187
	R: CTCTCGCACCCACTTCTTCTC		
CXCL6	F: CCAAGGTGGAAGTGGTAGCC	XM_005681937	149
	R: CTGGGCAATTCTTCCAACGC		
TLR-2	F: TTGACAAGAAGGCCATCCCC	NM_001285603	105
	R: AGAACGCTTCCTGCTGAGTC		
TLR-4	F: TTCAACCGTATCACGGCCTC	NM_001285574	127
	R: TGACCCACTGCAGGAAACTC		
MyD88	F: TTGAGAAGAGGTGCCGTCG	XM_013973392	187
	R: CAGACAGTGATGAAGCGCAG		
Tollip	F: CGACGTAGGCTTAGCGTGAA	XM_013976999	142
	R: CTGGTCTCACGCATCTACCG		
IRF3	F: TTGTGAACTCAGGGGTCAGG	XM_013971473	125
	R: TGGGCTCAAGTCCATGTCAC		
IFIT3	F: AAATTCTGAGGCAGGCCGTT	XM_005698196	127
	R: TTTCCCAGAGCCTCGACAAC		
NF-κB	F: CTGGAAGCACGAATGACAGA	XM_005681365	197
	R: GCTGTAAACATGAGCCGTACC		
GAPDH	F: CAAAGTGGACATCGTTGCCA	XM_005681365	197
	R: TGGAAGATGGTGATGGCCTT		

F, forward; R, reverse.

**Table 2:** Expression of inflammatory regulatory genes in control, TTO, LPS and TTO + LPS groups in in vitro incubated goat rumen epithelial cells.

Symbol	Treatment ^1^				SEM	*p*-Value
	Control	TTO	LPS	TTO + LPS		
*TLR-2*	1.00 ^b^	0.70 ^c^	3.15 ^a^	1.84 ^b^	0.30	<0.001
*TLR-4*	1.00 ^b^	1.09 ^a,b^	0.92 ^b^	1.44 ^a^	0.07	0.018
*CCL5*	1.02	0.94	1.78	1.55	0.15	0.105
*MYD88*	1.00	0.78	0.83	1.24	0.80	0.178
*TOLLIP*	1.01	0.64	0.84	0.98	0.06	0.049
*IRF3*	1.00 ^b^	1.19 ^b^	1.15 ^b^	1.45 ^a^	0.05	0.001
*NF-κB*	1.01 ^b^	0.73 ^b^	1.82 ^a^	1.67 ^a^	0.14	<0.001
*IFIT3*	1.01 ^a^	0.62 ^b^	1.20 ^a^	0.43 ^b^	0.10	0.002
*CXCL6*	1.00 ^c^	0.76 ^c^	12.31 ^a^	4.28 ^b^	1.44	<0.001

^a,b,c^^,^ Means in the same row with different superscripts differ significantly for treatment effect (*p* < 0.05). ^1^ GRECs were cultured in DMEM/F12 medium, DMEM/F12 medium supplemented with 0.05% TTO, DMEM/F12 medium supplemented with 1 µg/mL lipopolysaccharide and DMEM/F12 medium supplemented with 1 µg/mL lipopolysaccharide and 0.05% TTO for 24 h, respectively.

## Data Availability

Not applicable.
